# Acute Monocytic Leukemia With Histiocyte-Like Morphology and Trisomy 8: A Rare Diagnostic Challenge

**DOI:** 10.7759/cureus.109323

**Published:** 2026-05-20

**Authors:** Mehdi El Agal, Zakaria El Kodmiri, Zineb Guessous, Maryame Ahnach, Abdelkader Belmekki

**Affiliations:** 1 Faculty of Medicine, Mohammed VI University of Health and Sciences (UM6SS), Rabat, MAR; 2 Department of Hematology, International University Hospital Mohammed VI, Rabat, MAR; 3 Immunopathology-Immunotherapy-Immunomonitoring Laboratory, Faculty of Medicine, Mohammed VI University of Health and Sciences (UM6SS), Casablanca, MAR; 4 Faculty of Medicine, Mohammed VI University of Health and Sciences (UM6SS), Casablanca, MAR; 5 Mohammed VI National Medical Laboratory of Medical Analysis, Fondation Mohammed VI of Health and Sciences, Casablanca, MAR

**Keywords:** acute myeloid leukemia, aml-m5, hemophagocytosis, monocytic differentiation, trisomy 8

## Abstract

Acute monocytic leukemia (AML-M5) is a subtype of acute myeloid leukemia characterized by the proliferation of monoblasts and promonocytes showing monocytic differentiation. Although typical cytomorphological features are usually recognizable, unusual presentations may create diagnostic difficulties. We report a rare case of AML presenting with prominent histiocyte-like cells in the bone marrow associated with trisomy 8. Bone marrow examination revealed hypercellularity with two predominant cell populations consisting of monocytoid blasts and large histiocyte-like cells exhibiting abundant foamy cytoplasm and occasional hemophagocytosis. Cytochemical staining for myeloperoxidase was negative. Flow cytometry demonstrated an abnormal population expressing CD33, CD15, and CD4 with weak CD45 expression, while lymphoid markers were negative. Cytogenetic analysis identified trisomy 8 in the majority of metaphases. The patient received standard induction chemotherapy followed by consolidation therapy according to conventional AML protocols. This case highlights the importance of integrating cytomorphology, immunophenotyping, and cytogenetic findings to establish the correct diagnosis when atypical histiocytic-like features are present.

## Introduction

Acute myeloid leukemia (AML) represents a heterogeneous group of hematologic malignancies characterized by clonal proliferation of myeloid precursor cells in the bone marrow and peripheral blood [1]. Among its subtypes, acute monoblastic/monocytic leukemia (AML-M5) accounts for approximately 5-10% of AML cases and is defined by predominance of monoblasts and promonocytes showing monocytic differentiation [2].

In most cases, AML-M5 demonstrates characteristic cytomorphological and immunophenotypic features facilitating diagnosis [3]. However, atypical morphological variants may occur and represent diagnostic challenges. Rarely, leukemic cells may exhibit histiocyte-like morphology with abundant foamy cytoplasm and hemophagocytic activity, which may mimic histiocytic proliferations or reactive histiocytosis [4].

Such unusual features may lead to diagnostic confusion, particularly in settings where immunohistochemical markers for histiocytic differentiation are not available. Recognition of these atypical morphological presentations is therefore essential. We report a rare case of acute monocytic leukemia presenting with prominent histiocyte-like cells in the bone marrow associated with trisomy 8, emphasizing the importance of integrating morphological, immunophenotypic, and cytogenetic data for accurate diagnosis.

## Case presentation

A 38-year-old male with a history of type 2 diabetes mellitus diagnosed and poorly controlled under oral antidiabetic agents, bronchial asthma treated with inhaled corticosteroids and long-acting bronchodilators, an appendectomy at the age of seven, chronic tobacco use, and alcohol abstinence for two years was evaluated for progressive clinical deterioration.

The clinical history was marked by progressive onset of symptoms, including severe asthenia, pallor, lipothymia, and exertional dyspnea consistent with an anemic syndrome. The patient also reported hemorrhagic manifestations, including ecchymoses, gingival bleeding, and recurrent epistaxis. Additional complaints included intense bone pain and facial cellulitis involving the left maxillary region associated with sinus filling.

Initial laboratory investigations revealed severe pancytopenia with hemoglobin of 5.8 g/dL, white blood cell count of 2,420/mm³, neutrophils of 430/mm³, and platelet count of 85,000/mm³. Inflammatory markers were elevated, and renal impairment was noted with a decreased glomerular filtration rate. Uric acid levels were increased, suggesting high cellular turnover. The detailed laboratory findings are summarized in Table 1.

**Table 1:** Laboratory findings at admission GFR: Glomerular Filtration Rate

Parameter	Value	Reference Range
Hemoglobin	5.8 g/dL	13-17 g/dL
White blood cells	2,420/mm³	4,000-10,000/mm³
Neutrophils	430/mm³	1,500-7,000/mm³
Platelets	85,000/mm³	150,000-400,000/mm³
C-reactive protein	33.10 mg/L	<5 mg/L
Vitamin D	14.4 ng/mL	30-100 ng/mL
Albumin	33 g/L	35-50 g/L
Corrected calcium	84 mg/L	85-105 mg/L
Creatinine	24.12 mg/L	6-12 mg/L
Urea	0.46 g/L	0.15-0.45 g/L
GFR	32.22 mL/min	>90 mL/min
Uric acid	104 mg/L	30-70 mg/L
Prothrombin time	89%	70-100%
Activated partial thromboplastin time	34 s	25-35 s
Fibrinogen	2.22 g/L	2-4 g/L

**Figure 1:**
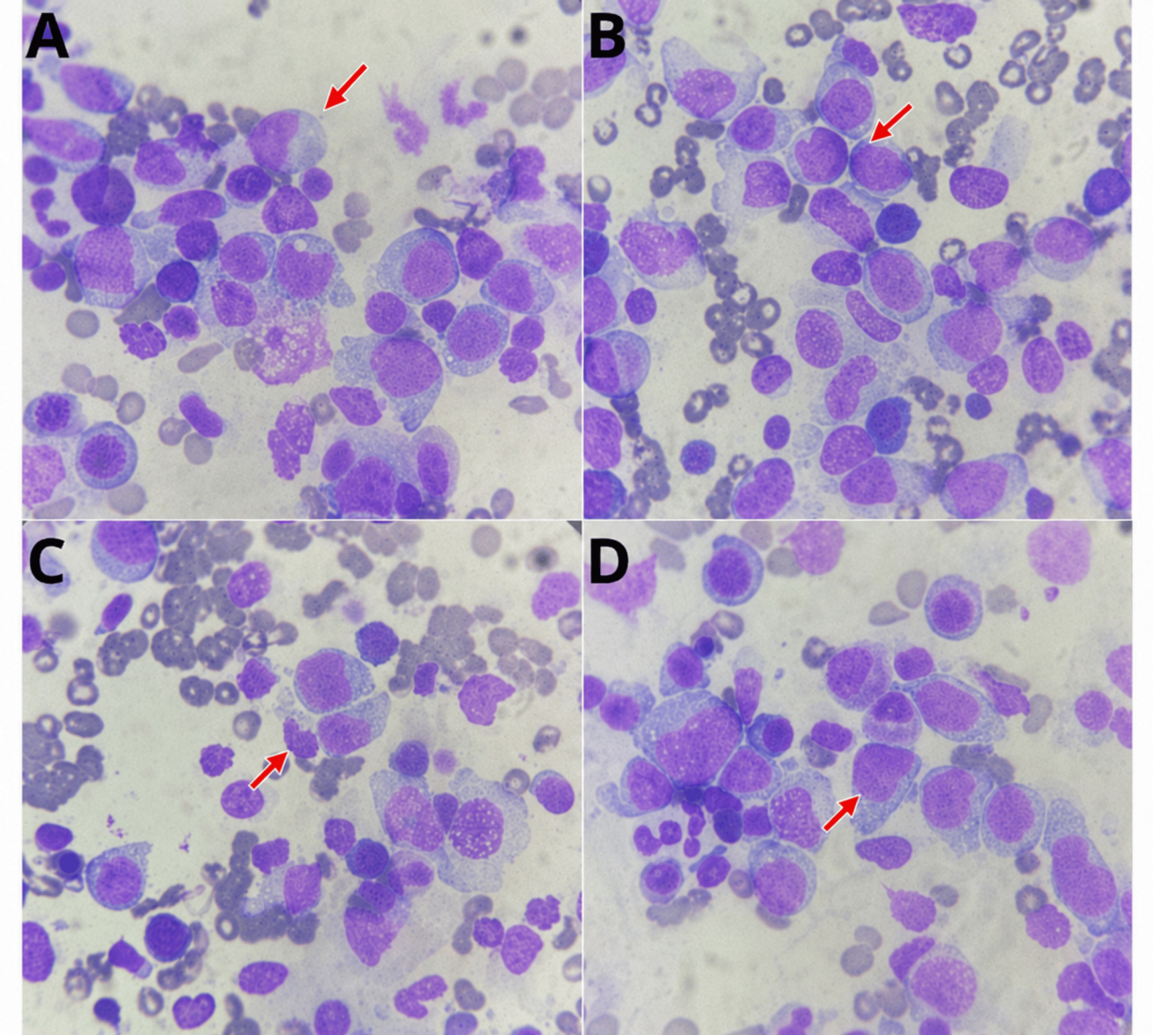
Bone marrow cytomorphology Bone marrow aspirate smears stained with May-Grünwald-Giemsa (MGG) revealed a hypercellular marrow infiltrated by monocytoid blasts characterized by abundant basophilic cytoplasm and irregular nuclei with prominent nucleoli (A). Some blasts showed cytoplasmic vacuolization and pseudopod-like projections suggestive of monocytic differentiation (B). A second population of larger cells with eccentric nuclei and abundant foamy cytoplasm resembling histiocytes was observed (C). Occasional hemophagocytosis was also identified (D).

Bone marrow aspiration demonstrated a hypercellular marrow infiltrated by atypical large cells. Two distinct populations were identified: monocytoid blasts with abundant basophilic cytoplasm, irregular nuclei, and prominent nucleoli, and a second population of large histiocyte-like cells with eccentric nuclei and abundant foamy cytoplasm. Occasional hemophagocytosis was observed. These cytomorphological features are illustrated in Figure 1.

Cytochemical staining for myeloperoxidase (MPO) was negative, consistent with monocytic lineage differentiation. Flow cytometry confirmed the presence of an abnormal leukemic population with weak CD45 expression, indicating an immature hematopoietic origin. The cells expressed myeloid and monocytic markers, including CD33, CD15, and CD4, supporting monocytic differentiation. Markers associated with the lymphoid lineage, including CD3, CD19, and CD79a, were negative, ruling out mixed-phenotype leukemia.

Immunohistochemical staining for histiocytic markers such as CD68 and CD163 could not be performed due to reagent unavailability in the laboratory at the time of analysis. Conventional karyotype analysis revealed a major clone representing approximately 70% of metaphases carrying trisomy 8 (+8), a recurrent cytogenetic abnormality in AML. A minor clone (30%) exhibited a normal karyotype, likely corresponding to residual hematopoiesis or a secondary leukemic subclone.

**Figure 2:**
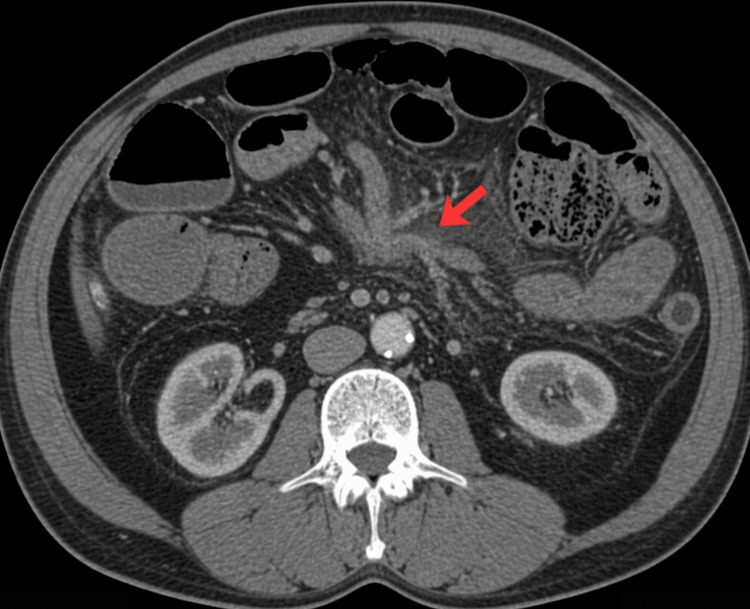
Abdominal CT scan showing mesenteric infiltration

Radiological evaluation revealed systemic involvement, as shown in Figure 2. A thoraco-abdomino-pelvic computed tomography scan demonstrated mesenteric infiltration, signs of pyelonephritis, and moderate bilateral pleural effusion. Abdominal ultrasonography showed aerocolia with abdominal distension.

The patient initially received supportive care, including antibiotic therapy, digestive symptomatic treatment with simethicone, and topical management of hemorrhoidal disease and anal fissure. Hematological management consisted of hypomethylating therapy with azacitidine (Vidaza) combined with cytarabine as first-line treatment. Subsequently, induction chemotherapy following the classical 3+7 regimen was initiated, consisting of idarubicin administered for three days and cytarabine for seven days. Close monitoring of hematological parameters, metabolic status, and infectious complications was maintained throughout the treatment course.

## Discussion

Acute monoblastic/monocytic leukemia (AML-M5) represents a distinct subtype of AML characterized by the proliferation of monoblasts and promonocytes with monocytic differentiation [5]. According to the World Health Organization classification, AML-M5 accounts for approximately 5-10% of all AML cases and is typically associated with characteristic cytomorphological and immunophenotypic features [6]. However, unusual morphological presentations may occur and occasionally create diagnostic challenges, particularly when leukemic cells exhibit atypical differentiation patterns [7].

In the present case, bone marrow examination revealed two morphologically distinct cell populations. The first consisted of monocytoid blasts, which displayed the classical cytological features of AML with monocytic differentiation, including abundant basophilic cytoplasm, irregular nuclear contours, and cytoplasmic vacuolization. These findings were further supported by the immunophenotypic profile, with leukemic cells expressing monocytic and myeloid markers such as CD33, CD15, and CD4, while lacking lymphoid markers. This immunophenotypic pattern confirmed the diagnosis of AML with monocytic differentiation.

The second cell population consisted of large histiocyte-like cells with eccentric nuclei and abundant finely granular cytoplasm with a foamy or ground-glass appearance. Occasional hemophagocytosis was also observed. Such morphological features are unusual in AML and may mimic histiocytic proliferations. The presence of these histiocyte-like cells represents the most distinctive aspect of this case and raises important considerations regarding differential diagnosis.

One of the main diagnostic challenges in such cases is distinguishing AML with monocytic differentiation from histiocytic sarcoma, a rare malignant neoplasm derived from mature histiocytes [8]. Histiocytic sarcoma typically exhibits strong expression of histiocytic markers such as CD68 and CD163 and lacks markers associated with myeloid blasts. In contrast, the leukemic population in the present case demonstrated a clear myeloid immunophenotype consistent with monocytic lineage. Although immunohistochemical staining for CD68 and CD163 could not be performed due to technical limitations, the presence of monocytic markers and the cytogenetic findings strongly supported the diagnosis of AML rather than a primary histiocytic neoplasm.

Another important differential diagnosis includes reactive histiocytosis or hemophagocytic activity associated with inflammatory or infectious conditions [9]. Reactive histiocytes may show hemophagocytosis and morphological features similar to those observed in this case. However, reactive processes are typically accompanied by preserved hematopoiesis and lack clonal cytogenetic abnormalities [10]. In the present case, the detection of a dominant leukemic clone with trisomy 8 supported the presence of a clonal hematologic malignancy.

The cytogenetic analysis revealed trisomy 8 as the major chromosomal abnormality, detected in approximately 70% of metaphases. Trisomy 8 is one of the most frequent cytogenetic abnormalities observed in AML, occurring in approximately 10-15% of cases. It is particularly associated with myeloid neoplasms showing monocytic differentiation. Although trisomy 8 is generally considered an intermediate-risk cytogenetic abnormality, its prognostic significance may vary depending on additional molecular alterations and clinical context [11]. Some studies have suggested that trisomy 8 may be associated with increased leukemic proliferation and potential resistance to chemotherapy, particularly in certain AML subtypes [12].

The coexistence of monocytoid blasts and histiocyte-like cells observed in this case may reflect the developmental plasticity of the monocytic lineage [13]. Monocytes and macrophages share a common progenitor within the myeloid lineage, and leukemic cells with monocytic differentiation may exhibit features resembling mature macrophages or histiocytes [14]. This phenomenon has been occasionally reported in the literature and may represent aberrant differentiation within the leukemic clone [15]. Such differentiation patterns may explain the presence of cells with abundant foamy cytoplasm and hemophagocytic activity in the bone marrow microenvironment [16].

Several reports in the literature have described AML cases with unusual histiocytic or macrophage-like features, although such presentations remain rare [17]. In these reports, leukemic cells exhibited morphological characteristics resembling histiocytes or macrophages, sometimes associated with hemophagocytosis [18]. These atypical features may lead to diagnostic confusion, particularly in settings where advanced immunophenotypic or molecular techniques are not readily available [19].

The present case, therefore, emphasizes the importance of an integrated diagnostic approach combining cytomorphology, flow cytometry, and cytogenetic analysis. Reliance on morphological evaluation alone could lead to misinterpretation of histiocyte-like cells and potentially delay the correct diagnosis. Accurate identification of AML with monocytic differentiation is essential, as it guides appropriate therapeutic strategies and clinical management.

Furthermore, this case highlights the role of bone marrow cytomorphology as a critical initial diagnostic tool, particularly in resource-limited settings where access to extended immunohistochemical panels may be restricted. Careful morphological analysis, supported by targeted immunophenotyping and cytogenetic testing, remains fundamental for establishing the correct diagnosis in complex or atypical presentations of AML.

## Conclusions

This case highlights a rare and diagnostically challenging presentation of AML-M5 with histiocyte-like morphology and hemophagocytosis. Such atypical features may mimic histiocytic disorders and lead to potential misdiagnosis. Recognition of these unusual morphological variants, particularly in association with cytogenetic abnormalities such as trisomy 8, is essential to ensure accurate diagnosis and appropriate therapeutic management. This report emphasizes the critical role of integrating cytomorphology, immunophenotyping, and cytogenetic analysis, especially in resource-limited settings.

## References

[1] Döhner H, Estey E, Grimwade D (2017). Diagnosis and management of AML in adults: 2017 ELN recommendations from an international expert panel. Blood.

[2] Arber DA, Orazi A, Hasserjian R (2016). The 2016 revision to the World Health Organization classification of myeloid neoplasms and acute leukemia. Blood.

[3] Salazar KL, Mosse C (2015). Normal karyotype in a case of acute myeloid leukemia with monocytic differentiation and hemophagocytosis by leukemic blasts. Lab Med.

[4] Roldán Galiacho V, Martin Martitegui X, Moreno Gamiz M, Arzuaga-Mendez J, Amutio E, García-Ruiz JC (2023). Acute monocytic leukaemia with histiocytic differentiation and erythrophagocytosis. Hematol Transfus Cell Ther.

[5] Sato S, Tamai Y (2022). Acute monocytic leukemia with histiocytic morphology in hematological remission after azacytidine and venetoclax therapy. Int J Hematol.

[6] Liu B, Li T (2020). Relapsed acute monocytic leukemia presenting as histiocytic morphology. Int J Hematol.

[7] Hastings A, Apperley JF, Nadal-Melsio E, Brown L, Bain BJ (2021). Acute myeloid leukemia with a severe coagulopathy and t(8;16)(p11;p13). Am J Hematol.

[8] Haferlach T, Schoch C, Schnittger S, Kern W, Löffler H, Hiddemann W (2002). Distinct genetic patterns can be identified in acute monoblastic and acute monocytic leukaemia (FAB AML M5a and M5b): a study of 124 patients. Br J Haematol.

[9] Collins NG, O'Connor HM, Lindsey KG (2020). Erythrophagocytosis by leukemic blasts in acute myeloid leukemia with a normal karyotype and no detectable mutations. Proc (Bayl Univ Med Cent).

[10] Liu J, Han W, Cai X (2022). Molecular genetic and clinical characterization of acute myeloid leukemia with trisomy 8 as the sole chromosome abnormality. Hematology.

[11] Mrózek K, Heerema NA, Bloomfield CD (2004). Cytogenetics in acute leukemia. Blood Rev.

[12] Grimwade D, Hills RK, Moorman AV (2010). Refinement of cytogenetic classification in acute myeloid leukemia: determination of prognostic significance of rare recurring chromosomal abnormalities among 5876 younger adult patients treated in the United Kingdom Medical Research Council trials. Blood.

[13] Mustapha MT, Özsahin DU (2025). Morphological analysis and subtype detection of acute myeloid leukemia in high-resolution. AI.

[14] Patterer V, Schnittger S, Kern W, Haferlach T, Haferlach C (2013). Hematologic malignancies with PCM1-JAK2 gene fusion share characteristics with myeloid and lymphoid neoplasms with eosinophilia and abnormalities of PDGFRA, PDGFRB, and FGFR1. Ann Hematol.

[15] Bruserud Ø, Selheim F, Hernandez-Valladares M, Reikvam H (2024). Monocytic differentiation in acute myeloid leukemia cells: diagnostic criteria, biological heterogeneity, mitochondrial metabolism, resistance to and induction by targeted therapies. Int J Mol Sci.

[16] Jain A, Gupta P, Gupta N (2021). Acute monocytic leukemia presenting as generalized lymphadenopathy and skin rash in a toddler: highlighting the clinicopathologic mimics. Diagn Cytopathol.

[17] Majluf-Cruz A, Sosa-Camas R, Pérez-Ramírez O, Rosas-Cabral A, Vargas-Vorackova F, Labardini-Méndez J (1998). Hemophagocytic syndrome associated with hematological neoplasias. Leuk Res.

[18] Sun Y, Blieden C, Merritt BY, Sosa R, Rivero G (2021). Hemophagocytic lymphohistiocytosis and myelodysplastic syndrome: a case report and review of the literature. J Med Case Rep.

[19] Farias MG, Freitas PA, Spagnol F (2021). Hemophagocytosis by blasts in a child with acute monocytic leukemia after chemotherapy. Rev Paul Pediatr.

